# The Effect of Two Types of Exercise Preconditioning on the Expression of TrkB, TNF-*α*, and MMP2 Genes in Rats with Stroke

**DOI:** 10.1155/2021/5595368

**Published:** 2021-04-12

**Authors:** Mostafa Teymuri Kheravi, Shila Nayebifar, Seyedeh Motahareh Aletaha, Sara Sarhadi

**Affiliations:** ^1^Department of Physical Education, Islamic Azad University Bojnourd Branch, Bojnourd, Iran; ^2^Department of Sport Sciences, Faculty of Educational Sciences and Psychology, University of Sistan and Baluchestan, Zahedan, Iran; ^3^Department of Exercise Physiology, University of Shahid Rajaee Teacher, Tehran, Iran

## Abstract

Despite the beneficial effects of exercise and physical activity, there is little knowledge about the effects of different types of physical activity on neural function. The present study assessed the effects of two types of selected aerobic exercises prior to stroke induction and characterized the expression of TrkB, TNF-*α*, and MMP2 genes in vivo. Forty male adult Wistar rats were exposed to aerobic exercises following randomization into four groups, including swimming + MCAO (Middle Cerebral Artery Occlusion) (*n* = 10), treadmill training + MCAO (*n* = 10), MCAO (*n* = 10), and control (*n* = 10). The swimming + MCAO group included swimming for 30 minutes each day, while the treadmill training + MCAO group program involved running for 30 minutes each day at an intensity of 15 m/min, for three weeks, five days a week. Neurological deficit was assessed using modified criteria at 24 h after the onset of cerebral ischemia. In the control group, the animals worked freely for three weeks without undergoing ischemia. The MCAO group also operated freely for three weeks after they underwent a stroke. Both training groups underwent ischemia after three weeks of training. TrkB, TNF-*α*, and MMP2 gene expressions were increased in the MCAO+ swimming training and in the MCAO + running training group compared to the control and MCAO groups, respectively. Preconditioning aerobic exercises significantly increased brain trophic support and reduced brain damage conditions in exercise groups, which support the importance of aerobic exercise in the prevention and treatment of stroke.

## 1. Introduction

Cerebral ischemia is a well-known pathological phenomenon that is associated significantly with high morbidity and mortality [1]. Injuries in the clinical condition of the disease include processes such as increased levels of neurotransmitters and immune-inflammatory activation. During ischemia, inflammatory responses lead to molecular and cellular events in the central nervous system. In this series of inflammatory changes, cytokines act as a central mediator in inflammatory waterfalls. Cytokines, such as the tumor necrosis factor-*α* (TNF-*α*), are proteins that express a wide range of physiological and pathological processes, including inflammation, cancer, autoimmunity, and infection [2]. TNF-*α* plays a key role in regulating the systemic inflammatory processes of inflammatory mediators of nerves and glia toxicity [3].

Activation of high levels of TNF-*α* has been observed in laboratory models with cerebral ischemia and in patients with stroke and large and small cerebral infarction [4]. After cerebral ischemia, nerve microglial cells undergo activated phenotypic changes and invade the affected area, exacerbating and maintaining damage through several mediators and pathogen pathways [2]. Among these mediators is the matrix metalloproteinase (MMP2). MMP2s are members of the zinc-dependent endo peptidases family, which are activated by many inflammatory signals. In particular, MMP2s can affect the integrity of the brain by damaging the endothelial function of the blood-brain barrier (BBB) and impairing the functional integrity of the vascular unit. These pathological events may open a door for environmental inflammatory cells to enter the brain, exposing nerve cells to attack by environmental immune cells [2]. Given the prevalence of stroke as one of the most important health problems worldwide, many studies have been conducted to identify the factors involved in the pathogenesis of this disease and its prevention and treatment [5].

Today, physical activity and exercise are considered a neuro-protective factor [6]. Exercise preconditioning (EP), which refers to the continuous and regular repetition of exercise before ischemia or disease, builds on a variety of methods, including promoting angiogenesis, inhibiting glutamate overactivation, protecting the BBB, and inhibiting apoptosis and inflammatory mediators, to protect the brain from stroke-associated events [7, 8].

Compared to other therapeutic methods, EP is a simple, noninvasive, and clinically feasible procedure. BDNF levels, for example, increase during exercise. The tropomyosin-related kinase receptor type B (TrkB), the signaling pathways associated with proliferation, differentiation, and neuronal residues, is formed subsequent to increase in this neurotrophin and its binding to related peculiar endogenous receptor [6, 9]. EP can also resist ischemic/recurrent damage by increasing the microcirculatory integrity of the brain through TNF-*α*, which increases the expression of integrins. It is shown that exercise enhances neurovascular integrity by regenerating integrins after a stroke [10].

Despite the beneficial effects of exercise and physical activity, there is little knowledge about the effects of different types of physical activity on neural function. Neurotrophin receptors, such as TrkB, and other proteins such as TNF-*α* and MMP2, as beneficial and harmful mediators in the stroke process, can be affected by a variety of physical activities. Three types of general sports activities are aerobic, anaerobic, and flexibility. Aerobic exercise refers to any physical activity involved with large muscle groups, and as a result, the body uses more oxygen than during rest time. The most important and common examples of this type of exercises include swimming and running [10]. Various results have been reported on the effects of exercise. As concerns with the effect of running on a treadmill on cerebral blood flow in animal models, for example, no significant changes in overall CBF were observed during moderate-intensity exercise. In contrast, running on a treadmill increases TrkB [11–13] and cerebral blood flow [14] in trained animals compared to sedentary animals. Awareness of the explicit mechanisms by which exercise can increase brain and heart endurance may encourage patients with high risk factors for cerebral and cardiovascular diseases to participate in appropriate exercise programs eagerly [10]. Awareness of the benefits of sports preconditioning may force more patients with ischemic stroke to accept exercise therapy [7].

## 2. Materials and Methods

### 2.1. Experimental Animals

This experimental study was performed with 40 male adult Wistar rats breed at the age of eight weeks and an approximate weight range of 200 to 250 grams purchased from the animal house of Faculty of Medical Sciences of Iran. The animals were kept for one week under identical conditions, including identical water and food (pellets), temperature and storage humidity (23°C ± 2 and relative humidity of 5 ± 55%), sleep cycle and light (12 : 12), sex, health status (all male and healthy), and free access to water and food. This study was conducted in accordance with the National Institutes of Health's guidelines and instructions for the maintenance and care of animals. All stages of exercise and research were conducted in line with the instructions of the Ethics Committee of the Bojnourd University of Medical Sciences with the code IR.IAU.BOJNOURD.REC.1398.017.

### 2.2. Study Design

After two weeks of familiarizing the animals with the environment and training protocol, the animals were randomly assigned into four groups, including swimming + MCAO (Middle Cerebral Artery Occlusion) (*n* = 10), treadmill training + MCAO (*n* = 10), MCAO (*n* = 10), and control (*n* = 10). In the control group, the animals worked freely for three weeks without undergoing ischemia. The MCAO group also operated freely for three weeks after which they underwent a stroke. Both training groups underwent ischemia after three weeks of training.

### 2.3. Exercise Protocols

The protocol for the swimming + MCAO training group included swimming for three weeks, five days a week, for 30 minutes each day at a water temperature of 32 ± 2° [15]. The protocol for the treadmill training + MCAO group involved running for three weeks, five days a week, for 30 minutes each day at an intensity of 15 m/min, a slope of 0, and a shock of 1.5 mA [8].

### 2.4. Induction of Transient Global Ischemia

To induce transient global ischemia, the animals were weighed and anesthetized with Ketamine (30 mg/kg) and Xylazine (10 mg/kg). Then, under sterile conditions, an incision was made in the anterior part of the neck to the size of 1-1.5 cm, and after determining, both common carotid arteries (CCAs) were exposed by bilateral neck incision. The CCAs were occluded with aneurysm clips for 30 minutes, and the clips were then removed to restore cerebral blood flow [16, 17].

### 2.5. Neurological Deficit Scoring

Neurological deficit was assessed using modified criteria at 24 h after the onset of cerebral ischemia, which included no deficit (score 0), forelimb flexion (score 1), forelimb flexion plus control of lateral push resistance (score 2), circling to one side (score 3), turning to one side plus reduced consciousness (score 4), and death or lack of awareness and mobility (score 5). Mice that showed a score between 1 and 3 were considered successful models after initial assessment [12].

### 2.6. Preparation of Samples for Real-Time

**Figure 1:**
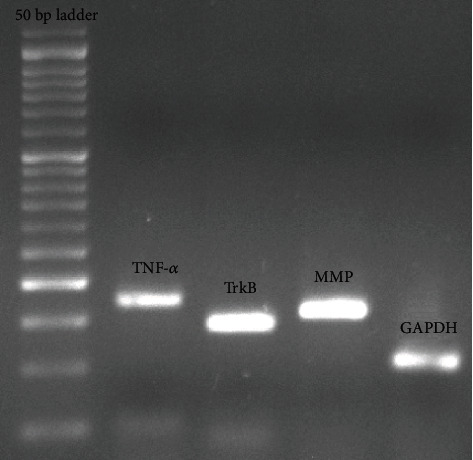
Real-time analysis of TNF-*α*, TrkB, MMP, and GAPDH as measured by Agarose gel electrophoresis.

Reverse transcription-polymerase chain reaction was used to reverse the expression of TNF-*α* and MMP2 genes in the brain tissue. At the beginning of the measurement, the brain tissue was beaten and slipped. Then, the extraction was performed according to the instructions using the RNA extraction kit protocol (Cat.No: EX6101 Sinagene Company). For reverse transcription (RT) reaction using cDNA synthesis kit, CatNo: YT4500 YektaTajhizAzma was used according to the instructions. The sequence of primers for each gene was as follows: TrkB, 5′-CACTCTTGGCGTAGATTGGC-3′ and 5′-AAGTAAACTCTCGGGGTGGG-3′; for TNF-*α*, 5′-CAATGGGCTTTCGGAACTCAC-3′ and 5′-TCAGGGAACAGTCTGGGAAG-3′; for MMP2, 5′-CATCAAATCGGACTGGCTGG-3′ and 5′-CAGGTGAAGGAGAAGGCTG-3′; and for GAPDH, 5′-CAACTTTGGCATCGTGGAAGG-3′ and 5′-AGGGATGATGTTCTGGGCTG-3′Figure 1. cDNAs obtained in Real-TimePCRIlluminar device and using SYBR green PCRMaster Mix Company of YektaTajhizAzma-Iran Company (Cat.No:PS4131) were examined. The performance of the primer bond with different genes was measured using standard curve drawing. The standard curve was plotted based on the concentration logarithm and threshold cycle (CT) for each gene.

### 2.7. Statistical Analysis

In the real-time section, the CT results were compared for data analysis. The results of gene expression were analyzed using the 2-DDCT method [18, 19]. The obtained data were analyzed in the SPSS 22.0 software using one-way analysis of variance and Benfroni post hoc test. The level of significance was set at *P* < 0.05.

## 3. Results

**Figure 2:**
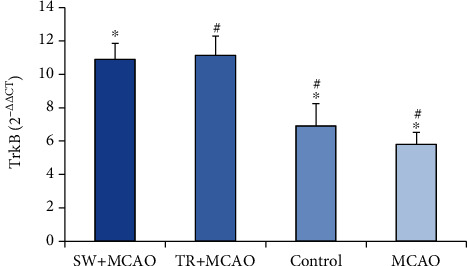
Average and standard deviation of TrkB gene expression. ^∗^TrkB indicates significance between MCAO +swimming training groups, control, and MCAO, and # shows significance between MCAO +treadmill training groups, control, and MCAO.

The results of ANOVA in the TrkB gene expression in the studied groups showed a significant difference (106/95, F (39, 3), *P* ≤ 0.001). The results of Benfroni's follow-up test showed that the TrkB gene expression in the MCAO+ swimming training group (11.06 ± 0.88) had been increased compared to the control group (0.61 ± 6.61) and to the MCAO group (5.80 ± 0.73). It was also increased in the MCAO + running training group (11.13 ± 1.15) compared to the control group and compared to the MCAO group (Figure 2).

**Figure 3:**
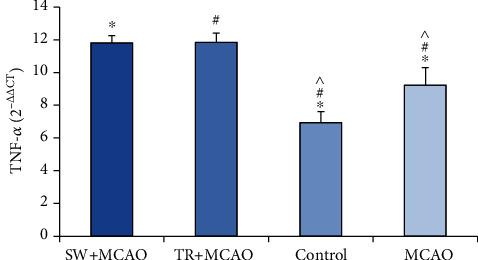
Average and standard deviation of gene expression. ^∗^TNF-*α* indicates significance between MCAO +swimming training groups, control, and MCAO, and # shows significance between MCAO +treadmill training groups, control, and MCAO. ^ shows significance between control and MCAO groups.

The results of ANOVA in the TNF-*α* in the studied groups showed a significant difference (111.17, F (39, 3), *P* ≤ 0.001). The results of Benfroni's follow-up test showed that the TNF-*α* gene expression in the MCAO+ swimming training group (11.81 ± 0.44) had been increased compared to the control group (0.67 ± 6.94) and to the MCAO group (9.23 ± 1.06). It was also increased in the MCAO + running training group (11.85 ± 0.55) compared to the control group and the MCAO group. The TNF-*α* gene expression in the MCAO was increased compared to control group (Figure 3).

**Figure 4:**
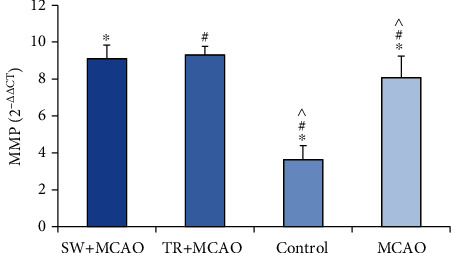
Average and standard deviation of gene expression. ^∗^MMP2 indicates significance between MCAO +swimming training groups, control, and MCAO, and # shows significance between MCAO +treadmill training groups, control, and MCAO. ^ shows significance between control and MCAO groups.

The results of ANOVA in the MMP2 gene expression in the studied groups showed a significant difference (105.53, F (39, 3), *P* ≤ 0.001). The results of Benfroni's follow-up test showed that the MMP2 gene expression in the MCAO+ swimming training group (9.11 ± 0.72) was increased compared to the control group (3.63 ± 0.76) and to the MCAO group (8.08 ± 16.1). It was also increased in the MCAO + running training group (9.31 ± 0.46) compared to the control group and compared to MACAO group either. The MMP2 gene expression in the MCAO group was increased compared to the control group (Figure 4).

## 4. Discussion

The structural, metabolic, and functional compatibility of the brain in response to physical exercise is interesting. Many studies report that physical activity of any kind and intensity causes changes, albeit small but constructive, in this organ [1]. Strong evidence supports the protective role of BDNF in reducing infarct volume and neural defects after stroke. BDNF cell proliferation increases phagocytic activity and inhibits microglia apoptosis in the brain.

BDNF activity is also able to regulate the TNF-*α* expression. Activated downstream paths by BDNF protect the cells from damage, such as serum starvation and glutamate toxicity. BDNF applies neuronal changes against ischemic damage by binding to the membrane receptor, p75 receptor, and TrkB receptor [18]. Research shows that physiological functions in animal exercises that occur in a variety of forms, including running on a treadmill or swimming in an enclosed pool, can affect stroke. Many studies show that both conventional exercise models of running and swimming can have positive and beneficial effects [20]. The results of our study showed that both exercise models of the present study could significantly increase the expression of TrkB gene in exercised animals. It is thought that exercise can facilitate and guide the mechanisms involved in improving some neurological diseases, including stroke, but the mechanisms that cause these changes are not fully recognized. TrkB signaling in pre- or postsynapse terminals leads to the regulation of several downstream genes. By activating the TrkB receptor and dimerization and phosphorylation of this receptor, totally three types of intracellular cascade signals are set up: (1) PLC-*γ* pathway, (2) PI3K pathway, and (3) MAPK pathway, which activates several downstream effectors. Ultimately, all three paths lead to the duplication of CREB (cAMP response element-binding protein) factor (the target gene for neurotrophins), and thus, the neurotrophin gene expression increases [6, 21]. However, the role of PLC-*γ* path due to the involvement of two factors, PKC (protein kinase C) and calcium, can be even more important because exercise plays an important role in calcium homeostasis. Activation of the PLC-*γ* leads to the launch of signals depending on IP3 (Inositol trisphosphate). IP3 in brain neurons leads to the rapid release of calcium from intracellular reserves and activates PKC, which leads to increased sensitivity of the contractile system and the release of calcium, followed by intracellular events such as proliferation, differentiation, and neuronal remnants [6]. Allen et al. showed that the expression of TrkB gene in the exercise group showed a significant increase compared to control by applying eight weeks of running on a treadmill five days a week for 30 minutes a day [22]. Using a TrkB receptor antagonist 12 (ANA-12), which blocks TrkB-dependent signaling path, the researchers showed that if the receptor did not work, the protective effects of exercise on nerves and cognition would not be applied. In 2012, Lee et al. [23] investigated the effect of running exercise on wheel running users on TrkB values in male Wistar rat hippocampus. The results showed that this type of exercise leads to improved cognitive function. In 2008, Liu et al. [24] showed that the expression of TrkB gene in the exercise group was much higher than the control group, and its level of expression was directly related to improving animal performance by four weeks of exercise on a treadmill in rats. Vedovelli et al. [25] state that the binding of BDNF to TrkB and the neurotrophin receptor P75 activates biochemical waterfalls that can lead to cell proliferation, survival, and plasticity. The results of a study conducted by Macaic et al. [26] showed that 28 days of walking on a treadmill increased the mRNA level of the BDNF gene, but it did not affect the expression of the TrkB receptor gene. The researchers say that the increase in BDNF has a greater impact on the recipient. Therefore, it is suggested that future studies measure the expression of BDNF gene and its TrkB receptor. This was one of the limitations of our research because measuring upstream and downstream pathways in a cellular mechanism can provide a broader view of stroke improvement.

Increasing the level of TNF-*α* during exercise is at least partly due to the release of activated platelets, known as TNF-*α* storage and release. This has been confirmed by the significant increase in BDNF level in serum, plasma, and platelets following exercise in healthy individuals. Some studies state that a prerequisite for exercise can resist against ischemic damage by increasing the brain microvascular integrity through TNF-*α*, which increases the expression of integrins [10]. Our results showed that TNF-*α* level increased in poststroke exercise groups. After cerebral ischemia, a series of inflammatory processes such as the penetration of neutrophils and monocytes and involvement of T cells and natural lethal cells can be observed. The first event of an inflammatory cascade is the microglia activation, followed by an increase in inflammatory cytokines such as TNF-*α*. TNF-*α* increases the toxic effects of other cytokines by synergistic ways, so it seems that these inflammatory cytokines regulate and induce inflammation in the nerve cell during cerebral ischemia [2]. But exercise and physical activity are able to change this role, and the results are reported to be inconsistent with the effects of exercise on TNF-*α* level. Zhou et al. [27] did not observe any change in serum TNF-*α* level after exercise by performing a 90-minute exercise protocol of running downhill, but its gene expression increased one-two weeks after exercise. They announced that TNF-*α* protein is mainly distributed in the cytoplasm and has a long-term expression up to two weeks after eccentric exercises. In our study, TNF-*α* levels were measured 24 h after ischemia. Due to changes in TNF-*α* levels immediately after exercise and then changes in the days after exercise, it is suggested that these changes be examined at different times of the day and even in the following weeks. Zhou et al. [28] showed that three weeks of exercise prerequisites reduced the concentration of TNF-*α* serum in the exercise group compared to the practice group. The researchers believe that the main mechanism of this effect is to control central and peripheral inflammatory cascades during cerebral ischemic injury [8]. Hung et al. [29] examined the effect of three weeks of running on a treadmill on TNF-*α* level, which showed that TNF-*α* level in practiced rats decreased more than the control group. Guo et al. [30] showed an increase in TNF-*α*, followed by a significant decrease in infarction volume by a three-week period of practice on treadmill and subsequent induction of a stroke. Some sources claim that skeletal muscle is an important source of several cytokines, often referred to as myokins. The release of these myokins into the bloodstream may be due to the systemic effects of exercise, including its protective potential. The downstream intermediaries of exercise may include TNF-*α* and kinases regulated by extracellular signals 1 and 2 (ERK1/2) [31]. One study showed that infarction volume was significantly reduced in rats with MCAo during three weeks of treadmill practice compared to the rats without practice, and this protection was associated with a gradual increase in TNF-*α* level in the brain [32]. In support of this finding, it has been shown that blocking the TNF-*α* signaling path with an anti-TNF-*α* antibody or inhibiting preischemic ERK1/2 activation destroys the protective effect in the brain [30]. Exercise regulates TNF-*α*. TNF-*α* produced in exercise plays a protective role through intracellular location, concentration, and interactions with other stimulated molecules. Mechanisms of the performance of TNF-*α* produced caused by exercise in reducing cases such as infarct volume, blood–brain barrier (BBB) impairment, loss of basal laminar protein, and poststroke MMP2 expression not only reinforce us in mechanisms under the influence of nervous protection caused by exercise but also provides a better insight about the therapeutic potential of TNF-*α* as a candidate for pharmacologic intervention [30].

Previous evidence shows that MMP produced in endothelial cells, microglia, and astrocytes is produced after the onset of permanent or transient ischemia in laboratory animals as well as in human patients. In the central nervous system, the advent of activated MMP2 is associated with changes in BBB permeability or the destruction of critical BBB components and the formation of vasogenic edema after focal ischemia. In addition, pharmacological inhibition of MMP2s after cerebral ischemia improves edema in cerebral edema. Our results showed that in the practiced rats, the rate of MMP2 increased compared to the stroke group. These results confirm the notion that exercise improves BBB function and subsequently reduces the MMP2 expression after stroke [30]. Sudden ischemia changes the relationships between endothelial cells, astrocytes, and ECM (extracellular matrix) integrity. Interactions of the astrocytic matrix of endothelial cells are the main stimulus for the onset of injury during a stroke. Therefore, an increase in ECM as a result of collagen IV resetting after physical exercise may play an important role in reducing brain edema and BBB permeability [30]. Wistar rats with ischemia were shown to have a significant reduction in the MMP2 expression after a period of running on a treadmill. The effects of running on the MMP2 expression were associated with improving cerebral blood flow and improving angiogenesis factors. As a result, therapeutic exercise improved long-term stroke outcome by mechanisms dependent on MMP2-VEGF related to improve cerebral blood flow [33]. Some studies have recorded a correlation between cytokines and MMP2 expression. The relation between increasing mRNA level of the MMP2 and TNF-*α* genes as well as an increase in their protein synthesis has been reported in brain-related diseases. Previous studies have shown that an acute increase in the TNF-*α* expression causes MMP2 through the activation of ERK. In contrast, some studies have shown that TNF-*α* regulation through exercise helps to reduce MMP2 [23]. Guo et al. [30] showed that the expression of MMP9 protein was increased compared to the control group in the stroke group. However, practice preconditioning reduced the MMP9 expression. This study also showed that inhibition of TNF-*α* or ERK1/2 in ischemic animals significantly increased MMP9 protein level.

## 5. Conclusion

In general, according to the results of the present study, it can be stated that two of the most common aerobic exercises have been able to make significant changes in cerebral ischemic indicators. Exercise can significantly increase brain trophic support and reduce brain damage conditions in exercise groups, which shows the usefulness of aerobic exercise in the prevention and treatment of stroke. However, our research may have limitations, and removing them may create more research opportunities to determine cellular and molecular mechanisms. In this regard, we can mention the duration and intensity of physical exercise, gender, old age, and ischemia. It is also recommended to examine other effective pathways, including the expression of neurotransmitters and other proteins such as glutamate, and the effect of exercise on these pathways, considering the background and variety of factors affecting the stroke process.

## Data Availability

Details are presented within the article in the forms of tables, text and images in results. Other data will be made available upon request.

## Additional Points

*Highlights*. Exercise can significantly increase brain trophic support and reduce brain damage. Two of the most common aerobic exercises have been able to make significant changes in cerebral ischemic indicators.
